# The Week's Work

**Published:** 1910-12-24

**Authors:** 


### The Week's Work.

### MR. SILVER'S BEQUESTS.

The particulars of the late Mr. Henry Silver's will, which we publish elsewhere in this issue (Personalia, p. 395), are interesting for various reasons. In the first place, these bequests are the largest which any one of the three hospitals has as yet received. In the second, they are positive evidence of the fact that the munificent charity which has hitherto been the main support of the voluntary system in this country is by no means extinct yet. It must be a matter of great satisfaction to the authorities of these three hospitals to know that they have received these gifts of money from a man who was intimately acquainted, through the personal service that he gave so ably and willingly during the best years of his life, with their work and needs, for it shows that he fully appreciated their difficulties, and that he had assured himself of the fact that they were eminently worthy of support. One of these institutions has lately had to bear what in our opinion was an entirely unwarranted attack on its efficiency, and it is gratifying to note that these attempts to belittle the good work that it has done and is still doing have been so signally negatived by this unusually large bequest. We trust that the authorities of the Great Ormond Street Hospital will lay stress on this fact. The "Dead Hand" has before now shown the public its duty, and we feel sure that in this case its influence will be very widely felt.

### CHRISTMAS AND THE HOSPITALS.

At Christmas-time the lay Press very kindly pays special attention to the needs of the voluntary hospitals, not only in placing prominently before the public the actual wants of these institutions, but also in "writing up" special phases of hospital work. For these attentions every hospital worker is cordially grateful. We have always held that the Press can do a great deal to encourage public interest in the hospitals, and we would be the last persons in the world to deprecate any attempt to make legitimate literary copy out of the many side-lights of hospital life that call for description and comment. But it is necessary sometimes to remind lay writers that accuracy and truth are the supreme essentials in such descriptions, and we regret to see in one of the evening papers an article which professes to give a description of the out-patient department of a large institution, and which is singularly idiotic and sentimental. Hospital workers who have perused the article will at once put their finger on the passages where the reporter has romanced to an extent which is more creditable to his generosity than to his powers of observation. These little blunders will only raise a smile with those who know the truth, but they give the public a false impression of the actual state of affairs, and are therefore calculated to do some harm. The *Star* on the same afternoon contained an excellent paper on the hospital patients' attitude of mind, which it is instructive to compare with the other. The one is a calm and telling summary of fact; the other an overdrawn, highly imaginative picture of what an out-patient department might be, but what it certainly under present conditions is not. In the days of "Calomel Curry" such things as our imaginative friend describes might have happened, but all of us who have worked in "outers," and who have been at some pains to observe what takes place there, know that they do not happen to-day.

### AN OUT-PATIENT DEPARTMENT INQUIRY.

H.S.H. the Duke of Teck, Lord Iveagh, and the Speaker of the House of Commons, Governors of King Edward's Hospital Fund for London, have announced their intention to appoint a Special Committee of Inquiry (as foreshadowed by his Majesty, when Prince of Wales, and President of the Fund) into the method prevailing in the London Voluntary Hospitals with regard to the admission of out-patients. The Committee will consist of Lord Mersey, who will act as Chairman, Lord Northcote, and the Bishop of Stepney. The Committee is ordered to consider and report generally as to the circumstances and conditions under which patients are admitted to the casualty and out-patient departments of the London Voluntary Hospitals, and especially as to what precautions are taken to prevent the admission of persons who are unsuitable, and as to whether adequate provision is made for the admission of such persons as are suitable; and to make such recommendations as may seem to them desirable. It is anticipated that the Committee will begin its sittings early in February, and we are asked to state that all communications intended for the Committee should be addressed to the Secretary, Mr. H. R. Maynard, King Edward's Hospital Fund for London, 7 Walbrook, E.C., and should be marked on the outside "Out-patients Inquiry Committee." The appointment of this Committee of Inquiry will be hailed with satisfaction by the hospital world in this country, since the subject which it is proposed to investigate is one that urgently demands attention. In view of recent discussions on hospital abuse the matter is particularly pressing, and the inquiry should yield valuable results.

### ANÆSTHETIC FATALITIES.

It is with no desire to embarrass or reflect upon the administration of Guy's Hospital that once more we feel it incumbent to offer comment on recent anæsthetic fatalities at that admirable charity. In 1907 the number of these catastrophes reached such a figure that public interest and apprehension were seriously aroused, and after The Hospital and other medical organs had discussed the situation sundry changes were made in the system of administering and teaching anæsthetics. The result was that the percentage of fatalities during anæsthesia at once dropped down to, or even below, that average which, in view of the desperate plight of some of the patients admitted to a general hospital, it is perhaps hopeless ever to eliminate entirely. This happy state of affairs proved at once the weakness of the old system and the efficiency of the reforms, which were instituted, be it remarked, entirely from within. Lately the open ether method has been introduced at Guy's, probably with the idea that this is the safest method to teach students, who, as they reach the rank of qualified house officers or as general practitioners, may have to give anæsthetics unaided and unsupervised. The advantages claimed for the open ether method have always included, first and foremost, absolute safety during anæsthesia: it was said, when the method was introduced, that it was impossible to cause death by over-dosage. Such a claim on behalf of any system of anæsthesia in which preliminary dosage with morphia plays the part which it does with open ether demands careful scrutiny. That it is not by any means true is shown by the fact that there have been three deaths at Guy's Hospital this year under open ether alone. This is quite apart from sequelæ, such as ether pneumonia, which is more frequent than some advocates of open ether believe.

### THE OPEN ETHER METHOD.

The last of these cases is reported in the daily press of December 13. The inquest concerned the death of a woman who was being operated on by Mr. Arbuthnot Lane for chronic constipation due to an adhesion following an operation for appendicitis. The patient, according to the medical evidence, had undergone several previous operations under anæsthesia. The death took place about an hour after the commencement of the operation, and the administrator was one of the resident staff, newly qualified, whose experience amounted to between one and two hundred cases. A preliminary dose of morphine had been given, as is usual with this method of exhibiting ether. The only other material detail to be gathered from the reports is that the pathologist, Dr. Spilsbury, found the status lymphaticus to be present, and attributed to this (according to the *Daily Telegraph*) some influence on the fatal issue. How this is to be reconciled with the fact that the woman had already passed successfully through several operations, including one for appendicitis, the pathologist is not reported to have explained. So much for the evidence. What strikes attention at once is the fact that the operation was one of convenience, not of urgency; and that it must have been appointed beforehand, probably two days at least. It seems strange, therefore, that none of the ten visiting anæsthetists, and not even the resident anæsthetist, should have been in attendance. To anæsthetise a patient for the intestinal operations by which Mr. Lane has made himself famous must be no light task, nor one to be undertaken by the first available qualified man who happens to be in the theatre. In other words, we are compelled to note that a reversion to the old slackness of three years ago seems to have set in. We shall be glad to hear that this is not so, but the evidence seems to favour the supposition. The other important point for the profession is that three deaths have occurred in one year in one hospital from the use of an anæsthetic which some anæsthetists still maintain to be so absolutely safe that it cannot directly cause death. The truth is, as Dr. Hewitt has long maintained, that the personality, care, and training of the administrator count for more than the particular drug employed to produce anæsthesia, or the particular method by which it is exhibited.

### INCORPORATED ASSOCIATION OF HOSPITAL OFFICERS.

We are indebted to the *Hospital Gazette* for a full account of the dinner of the above Association, which certainly marks a step forward not only in the usefulness but in the importance of this body. It is capital to find the President, Mr. Walter Alvey, alive to this fact, and anyone who had the pleasure of hearing or reading his speech will not be surprised at the very high praise it excited from Sir Savile Crossley and Lord Claud Hamilton. A man with a policy is always a man worth listening to, and Mr. Alvey has done good work in nursing the Association to its present incorporated condition; he showed how its existing lines may develop in a statesmanlike fashion, and produce a crop of results, which those who criticised its foundation will be bound to accept as of the first value. For instance, Mr. Alvey pointed out that it has been only by the solidarity of which the Association is the organised expression that the King's Fund has been placed in a position to judge the managerial point of view; the hospital managers, by meeting together as a body, have been able to contrive a general compromise on questions of detail which has gained for them a hearing such as would not have been accorded, with the best will in the world, to sporadic or to individual suggestions. As regards the future, Mr. Alvey mentioned that the possibility of a combined purchase of food and stores for the hospitals was under examination; and this is the sort of thing that really is high politics, or, in other words, constructive work. We send our readers to the Association's official organ for details and a full report, only adding here that we think Mr. Walter Alvey should complete his work by sending to the technical and medical journals reports of his Association's doings, since it is largely by his work that the Association has gained enough prestige and importance to warrant this publicity. The Association, having won a hearing, should be shy no longer, but should come out boldly into the papers and make its opinions felt.

### THIS WEEK'S DRUG MARKET.

Mainly owing to the near approach of the Christmas holidays business in the drug market is much quieter, and no noticeable improvement is likely to set in before the second or third week of the New Year. High prices have again been paid for gum asafœtida of fine quality, and extreme rates have been paid for castoreum. Camphor seems likely to tend somewhat dearer. Jalap is rather lower in value. Cascara sagrada is in better demand, and prices may advance.

